# Exploring mental health challenges and coping strategies in university students during the COVID-19 pandemic: A case study in Dhaka city, Bangladesh

**DOI:** 10.3389/fpubh.2023.1152366

**Published:** 2023-05-03

**Authors:** Masum Billah, Shannon Rutherford, Sadika Akhter, Mumita Tanjeela

**Affiliations:** ^1^Department of Sociology, East West University, Dhaka, Bangladesh; ^2^School of Medicine and Dentistry, Griffith University, Gold Coast, QLD, Australia; ^3^School of Health and Social Development, Deakin University, Melbourne, VIC, Australia

**Keywords:** mental health, coping strategies, social support, resiliency, students, Bangladesh

## Abstract

**Background:**

Mental health challenges have emerged worldwide during the COVID-19 pandemic. University students experienced changes in their lifestyles, academic life, family relationships, earning capacity, and support systems. This study explores the common mental health challenges in university students and their coping strategies using social support in the first wave of lockdowns in Dhaka city in 2020. By learning from young people’s impacts and coping responses, we can help build an improved strategy for future events of this magnitude.

**Methods:**

A qualitative study design was employed to conduct 20 in-depth interviews and two focus group discussions with students from purposively selected three public and three private universities in Dhaka city and five key informant interviews with different stakeholders. We used inductive reflexive thematic analysis and applied six phases of the thematic analysis. Codes retrieved from two differently prepared codebooks were merged and compared to identify themes for a fair interpretation of the underlying data. Data were manually indexed, summarized, and interpreted to categorize codes into sub-themes leading to themes.

**Results:**

Financial constraints, academic pressure, learning resources shortages, losing confidence, relationship breakup, excessive internet dependency, and traumatic experiences challenged the mental health conditions of the students unevenly across universities during the COVID-19 pandemic. Expressed mental health well-being impacts ranged from anxiety, stress, and depression to self-harm and suicidal ideation. Family bonding and social networking appeared as robust social support mechanisms to allow students to cope with anxiety, stress, and depression. Partial financial subsidies, soft loans to purchase electronic resources, faculty members’ counseling, and sessional health counseling contributed to minimizing the mental health impacts of COVID-19.

**Conclusion:**

Mental health is still not a resourced area of health and well-being in Bangladesh. Concentration on developing strong social support and improving increased financial subsidies, including learning resources, can be effective in assisting students in coping with the common mental health burdens during pandemic periods. A national intervention plan should be immediately designed and implemented by engaging different stakeholders including healthcare professionals and establishing effective mental healthcare support centers at universities to avoid immediate and prolonged negative mental health impacts.

## Background

The world has struggled to restrict the spread and health impacts of the SARS-CoV-2 virus and its rapid mutations. Bangladesh experienced a period of socio-economic stagnation due to a lockdown between March and May 2020 when, among other large societal changes, students could not attend university campuses. This lockdown policy was part of a multifaceted strategy that included full or partial lockdown periods, identification of COVID-19 zones, social distance or travel restrictions, and mass COVID-19 testing. Bangladesh identified the first three cases of COVID-19 on 8 March 2020, and the government decided to close all educational institutions to curb the spread of the virus. According to the WHO (1), 60% of students suffered from nationwide closures of schools, colleges, universities, and other educational institutions. These closures worldwide affected students’ academic growth and mental well-being (2). The young population aged 15–24 represents 20% of around 160 million people in Bangladesh (3).

Infectious disease outbreaks and the ways they are managed to restrict transmission affect people’s mental health. All around the world, researchers have reported high levels of mental health impacts in students during the COVID-19 pandemic (4, 5). One Chinese study in Hubei province showed that 24.9% of undergraduate medical students experienced anxiety. Of these students, 0.9% had severe anxiety, and 21.3% had mild anxiety (6). Pronounced levels of stress and significant psychological distress were also exhibited by students in France, Canada, and the United Kingdom (7–9). Likewise, individuals aged 17–20 experienced stress and anxiety due to the pandemic in Tehran, where prolonged confinement at home and overuse/overdependence on the internet and social media platforms during the pandemic were reported to negatively influence the resilience of the age group (10). A large longitudinal study that spanned the pandemic in university students in China revealed that the prevalence of anxiety and depression symptoms above the standardized threshold increased by 2.62 and 20.63%, respectively, to 4.06 and 22.09% following the pandemic (11). Meanwhile, another study in China just after 1 month of pandemic in 2020 showed that college students who had extreme fearful experience of the outbreak, those living in the worst-hit locations, and graduating or final year students had the highest risk of developing post-traumatic stress disorder and/or depression (12). Other studies reveal that the cumulative psychological pressure caused by the pandemic and quarantine increased anxiety and depression in the general population, particularly among university students in Shenzen in, China (13–15).

Coping strategies are multifaceted, covering a broad spectrum of human emotions, perceptions, and experiences. Social connection, social support from people, institutions, and their families, and religious beliefs were reported to serve as first-line coping pathways against longstanding collective trauma and stressors during the COVID-19 pandemic in the United States, Sweden, China, and Iran (16–20). These studies showed a potential linkage between individual-level coping and social connectedness. Social support and attachment have been cited across five studies for resilience building as a precursor to reducing anxiety, stress, and depression. Only high levels of social support may compensate for lower resilience (21–23). Similarly, trait resilience, social participation, and trust were associated with lower stress and distress levels (24). Resilience building is recognized and made evident through embedding resilience in the social structures, social processes, and the ideological and societal expectations of social groups (25). Overall, resilience is often interpreted as a buffer capacity for recovering one’s previous state (26). Considering the aforementioned, resilience building is often used by individuals, groups, and organizations to “bounce back” and persist during the aftermath of a crisis (27). Hence, it is evident that building resilience is essential for positive mental well-being amidst COVID-19.

Following a broad suite of COVID-related restrictions on March 17, 2020, the partial opening or closing of educational institutions challenged students’ mental well-being (28). The COVID-19 pandemic remains a period of turmoil and uncertainty. It has challenged us to understand better what resilience and resilience building is and how they can develop or be challenged in individuals and societies. Students experienced inevitable changes in the trajectory of their lives, including lifestyles, relationships with family and community, and reliance on support. A considerable number of quantitative studies revealed the association between different independent variables and outcomes of interest for mental health challenges in students during the COVID-19 pandemic. As mental wellbeing associated with several non-public health and environmental issues, we felt to develop a better understanding of the underlying factors and coping strategies of students to get a holistic picture of mental health landscape. To our best knowledge, this is the first qualitative study we conducted to get a deeper understanding of the mental health challenges in university students and their coping strategies during the pandemic. As the pandemic-ordained lockdown paved the way for virtual learning, students struggled to cope with the new normal situation. To better understand resilience building and interactions with social support this is key to explore how students who faced adversity during this time were impacted and what constructs helped to minimize the negative and maximize any positive impacts. This study aimed to investigate the common mental health challenges in university students and their coping strategies using social support during the COVID-19 pandemic in the first wave of lockdowns in Dhaka city in 2020.

## Methods

### Study design and data collection

A qualitative research design was employed to achieve the overall objectives of the current study. We collected qualitative data through in-depth interviews (IDIs), focus group discussions (FGDs), and key informant interviews (KIIs) to explore mental health challenges and coping strategies using social support. A group of researchers having qualitative research expertise conducted online interviews and offline FGDs with university students. Individual consent forms and separate open-ended interview guidelines were used to conduct IDIs, FGDs, and KIIs with the study participants. All the qualitative data collection tools had been finalized after piloting at the field level. Interview questions that contained in the checklist for IDIs and guide for FGDs and KIIs are presented in the Table 1.

**Table 1:** Interview questions.

Examples of interview questions	
In-depth interview	1. What did you feel different during lock down periods?
	2. Could you please explain the socio-economic conditions and academic life that affected you and your family?
	3. Could you please explain your mental health conditions during last 1 year (elaborate)?
	4. Is there any noticeable change in your life style?
	5. Could you please explain the strategies you adopted to cope with your mental health conditions?
	6. How do you find the importance of social support to cope with the situation?
	7. Could you please explain any supportive mechanisms you applied to cope with mental health problems like anxiety, stress, and depression?
	8. Did you get any institutional support to cope with your academic life and mental wellbeing (material or non-material like financial subsidy or mental health support counseling)?
Focus group discussion	1. What were the circumstances that you faced during the COVID 19 lockdown period?
	2. How did COVID 19 affect different aspects of you lives?
	3. What were the strategies you adopted to cope with the changing situation?
	4. What supportive mechanisms did you use to cope with the new normal situation or barriers?
	5. Were there any obstacles to using the supportive mechanisms that were available?
Key informant interview	1. Could you kindly explain the impacts on students that you observed during COVID 19 pandemic?
	2. Could you kindly describe the observed or reported obstacles for students regarding online education system throughout the country?
	3. How would you rate the role of public and private universities to impart online education and mental health support and counseling?
	4. Could you kindly tell us whether students had been well placed to adapt with the new normal situation due to COVID-19?

For online data collection, the study team used a Google meeting link for each interview separately by turning on the recording option to store the entire conversation online. Our research assistants prepared transcription after receiving the interview audio from investigators. Each recorded interview conducted in Bengali was automatically downloaded to facilitate transcription from audio to written scripts. We transferred the written narratives later into English. We labeled each respondent with a hypothetical number to guarantee anonymity and confidentiality. The investigator and co-investigators closely observed the transcription process and provided their valuable comments to go through a re-check process if anything looked missing or less prioritized in the transcription. For FGDs, we organized offline meetings to collect qualitative data. The online and offline data collection was between July and October 2020. This study was approved by the ethical review committee board in the East West University, Dhaka, Bangladesh. Ethical guidelines were followed throughout the entire research. As data collection was online for IDIs and KIIs, the investigators sought the participant’s verbal consent recorded in audio. For FGDs, the participants consented to our study, putting signature with personal details in the predesigned participant information sheet. Before seeking consent, all the participants were briefed about the nature and scope of the study. It was a promise to keep their personal information confidential. We strictly maintained the anonymity and flexibility of participants. Personal information was kept private so individuals could not be identified in any reported findings.

### Sampling

The target population was university students aged 19–24 years. This study followed purposive sampling to choose study participants. To collect qualitative data, we conducted 20 IDIs, two FGDs, and five KIIs. IDIs were conducted with students from three public and three private universities in Dhaka city. The purposively selected universities are approved by the Bangladesh University Grants Commission (UGC). In order to recruit participants for IDIs and FGDs, we followed inclusion and exclusion criteria. We included full time university students of 19–24 years who were enrolled in the selected universities in Dhaka city during interview period. On the contrary, we excluded students who were below 19 and above 24 years. We also excluded part time or working program students who attended evening programs. College and medical students and those students who attended universities outside of Dhaka were excluded from the study sample. We recruited university students for in-depth interview and focus group discussion using our own local connections. Faculty members and students of different universities, both former and current ones help to get the desired number of participants for the interviews. Before finalizing the recruitment of participants, we repeatedly crosschecked the inclusion and exclusion criteria of the pre-designed participant recruitment protocol and proof of university identity card, provided that they are currently enrolled in the program. We checked their valid student identity card and confirmed the accuracy of their engagement in the study. We conducted interviews with students asking general questions about the lockdown experiences, mental health conditions, and coping strategies during the COVID-19 home stay periods. Probing and follow up questions were in place to continue with interviews. Theoretical saturation was achieved based on emerging new themes in the subsequent interviews. It took 20 individual in-depth interviews and two focus group discussions of 10 students to reach theoretical consensus where no more new themes appeared. The full range of constructs that build the theory was completely represented by the collected data and it confirmed the achievement of theoretical saturation for this study (29). Two FGDs were organized with a group of 10 students, males and females, in equal numbers in a public and a private university independently. KIIs were conducted with psychologists, sociologists, and the secretary of the UGC. Each IDI and KII interview was between 30 and 40 min. For each FGD, the period was around 80 min.

### Data analysis

We analyzed data by utilizing inductive reflexive thematic analysis. We followed the recursive process and applied six phases of thematic analysis (30). The two investigators (MB, MT), trained in qualitative data analysis, primarily familiarized themselves with the transcribed data and independently developed a set of codes. Data were coded to categorize the key ideas, and categorical aggregation was employed to analyze and interpret the existing data (31, 32). Data were manually indexed, summarized, and interpreted to categorize codes into sub-themes leading to themes. Codes were framed by reading and re-reading narratives from IDIs, KIIs, and FGDs simultaneously. Codes retrieved from two differently prepared codebooks were merged and compared to identify sub-themes and themes, respectively. Two investigators (MB, MT) repeatedly consulted the codes followed by a series of meetings and reached consensus on selecting sub-themes and themes. Twenty-three codes were generated from the descriptive texts. These codes were categorized into three themes: mental health challenges, coping strategies, and institutional responses. A total of six sub-themes derived from the three general themes are shown in the Table 2. We (MB, MT) followed a back and forth policy to read the written transcription until theoretical saturation was achieved. In order to establish rigor in the findings, we applied three categories of triangulation: (i) method triangulation, (ii) data source triangulation, and (iii) investigator triangulation (33, 34). Firstly, for method triangulation, we employed three methods: in-depth interview, focus group discussion, and key informant interview to collect data for this study. Secondly, we collected data from three different types of study participants that assisted to reveal and identify similar findings. Thirdly, we adopted investigator triangulation process by engaging more than one researcher to conduct interviews, coding, and data analysis which strongly enhanced the clarity of the study findings (33).

**Table 2:** Themes of the study findings.

No of themes	Themes	Sub-themes
Theme 1	Mental health challenges	Stress, anxiety, and depression
		Serious mental illness and suicidal behavior
Theme 2	Coping strategies	Individual and social support at the family level
		Individual and social support at the community level
Theme 3	Institutional responses	Financial subsidies and soft loan incentives
		Mental health support and counseling

## Results

### Theme 1: Mental health challenges

The first theme shows mental health challenges developed in university students in Dhaka city. This theme comprises of two sub-themes such as anxiety, stress and depression, and serious mental illness and suicidal behavior. Lockdowns, mobility restrictions, and closures of universities indisputably affected mental well-being during the COVID-19 pandemic.

#### Anxiety, stress, and depression

Anxiety was commonly self-reported by students. Social media stories, uncertainty about life, news about COVID-19, and browsing face-book resulted in anxiety. Isolation, loneliness, fear of infection, boredom, uncertain academic life, and fear of going outside were reported as contributing to anxiety in students. One first-year student explained:

*“I felt restricted and isolated during lockdown periods. I had no friends; I could not explore my campus. This is not how I imagined my university life. I did not want to be hooked to my phone as it is a type of addiction…at the outset, I felt frustrated and angry but mainly anxious. I felt like I was trapped I could not describe my feelings. I stopped going outside as I did before the pandemic”* (IDI 3, Male, 22)

Another shared her feelings and the impacts of her emotions on her studies:

*“I started being irritated due to the restrictions, and online classes drained me out. I lost my focus on everything, and I had no spirit. For instance, I was not serious about anything. I started taking things lightly. I could discuss everything with my faculty offline, but now it is difficult to communicate online”* (IDI 2, Female, 24)

The majority of students also experienced mental stress due to financial challenges. Income loss of older adults, salary reductions in earning household members, and closures of family businesses created financial stress for participants. Moreover, loss of tuition fees, loss of part-time work and teaching assistantship, and difficulty in paying semester fees were identified as socio-economic hurdles for students. Overall, participants experienced changes in their lifestyles. Losing confidence, losing interest in online classes, losing concentration on studies, brain stagnation, and addiction to virtual platforms contributed to increased mental pressure for students. One participant talked about his feelings:

*“Over the days, my confidence has dropped unexpectedly. The ongoing online education has a great limitation which I feel. Once I used to attend debate competitions, and even I participated in several competitions at the national level. To my best knowledge, my level of excellence and skills has tremendously decreased over the days”* (IDI 1, Male, 21)

Another participant identified the losses of social interaction:

*“I used to participate in many co-educational activities during offline classes. Since I started online classes, I could not participate in everything due to the stress of my studies. I used to spend time in clubs when the classes were over. I enjoyed those face-to-face conversations that were available offline. Suppose I turn on the computer for some academic purpose, but after turning it on, I go to YouTube. I try to drive myself when I feel upset. I spend my spare time watching Netflix”* (IDI 4, Male, 21)

Depression was reported in university students during the COVID-19 pandemic. Most participants felt depression due to their and family members’ infection and the death of family members and relatives due to COVID-19. Aside from academic pressure, loss of physical, social connection, loss of freedom, sleep problems, and personal relationship challenges were also mentioned as contributing to mental well-being. One participant expressed her feelings:

*“My relationship turned out challenging due to the distance from lockdowns. I went through a breakup this year, and COVID-19 was unexpectedly responsible. Before getting infected with the coronavirus, I did not face many problems. After my beloved uncle’s death and my illness, I mentally broke down and experienced psychological difficulties. However, I felt burdened to continue the relationship with my partner and sought mental peace”* (IDI 17, Female, 23)

In addition, some of the participants reported associated physical health problems, such as headaches, migraine, and weight gain due to depression. One participant said his views:

*“Within the first three months of COVID-19, my coupling relationship broke down and I became emotionally frustrated. I could not sleep well. There was no way to make my mood better. I felt a lot of headaches due to depression. Although I used to suffer from depression due to personal reasons, now I am depressed about the education sector, and I am restless with back-to-back exams, quizzes, and assignments”* (IDI 1, Male, 21)

#### Serious mental illness and suicidal behavior

A few students experienced severe mental health impacts. One respondent resorted to self-harm, and another exhibited suicidal tendencies. Furthermore, two respondents experienced a worsening of existing bipolar disorder, and hallucinations, respectively. Doctors detected their mental health illness before the start of the COVID-19 pandemic, and they were under medical surveillance. During the COVID-19 lockdown period, they felt the severity of the diagnosed mental health illness. One FGD participant expressed her feelings:

*“I once cut off my hair as I could not express myself or socialize. I lost concentration in reading. My father also triggered me as he mentioned how he was ashamed to mention to his friends that I study at a private university. I retaliated by shouting. I was so frustrated that I took the scissors and cut off my hair impulsively”* (FGD, Female, 23)

Another study participant addressed his expressions:

*“At first, my mother was mistreated, and we all had trouble breathing at night. Second, I used to do politics on campus. The political crisis is always there. During the lockdown, I became panicked by sitting at my home. Everyone was giving consoles online, which did not work for me. By July 2020, I decided that I might commit suicide. Later I realized that it was the wrong decision”* (IDI 18, Male, 22)

### Theme 2: Coping strategies

The second theme reveals several coping strategies for students during COVID 19. Their coping strategy varied, and they became dependent on multiple supports at the family and community levels. When asked about survival strategies or mechanisms, participants reported that they constantly relied on their individual and social support systems to cope with mental health challenges during lockdown or homestay periods. Students spent COVID-19 days playing indoor games, cooking, drawing pictures, watching drama and movies, browsing the internet, and connecting with family members through social media networks. Their social support involved bonding relations with family members, cousins, and close relatives at the family level, neighbors, friends, teachers, and educational authorities at the community level. This theme consists of two sub-themes such as individual and social support at the family level and individual and social support at the community level.

#### Individual and social support at the family level

Playing indoor games, cooking, gardening, listening to music, drawing pictures, and caring for pets were reported as coping strategies for university students. One participant addressed his viewpoints:

*“As I said, I attended many courses online. I watched movies and TV series, listened to music, drew pictures, wrote poems, and tried to play the guitar during lockdowns. Our life changed randomly; it seemed like surviving to remain fit…We cannot ignore mental well-being. Due to class pressure online, I had no time for overthinking. I tried to remain positive”* (IDI 10, Male, 22)

Family bonding was reported as social support to cope with pandemic conditions. Students spent quality time with their family members. Their siblings became their best friends. A few respondents who lived in a close-knit family with extended family felt comforted compared to those with small nuclear family members. One study participant expressed her feelings:

*“I got mental support from my family and relatives. I have some cousins, and I always share my thoughts with them, and they share too. If I talk about social connectedness, I used social media, which has helped me in this purpose. Through texting and video call, I maintained contact with my relatives and cousins”* (IDI 20, Female, 21)

Another respondent shared her feelings on the benefits of online connections, but was able to recognize the potential negative impacts of connecting this way:

*“As I mentioned, I went to our old family home, which didn’t help. I enjoyed watching Korean dramas. I loved to talk to unknown people via social networks without disclosing my identity. It is easier to express yourself to people who don’t know you. No matter how much we accuse Facebook of being a waste of time, it helps us forget many things through memes, videos, and trolls. There should be a sense of control. Without utilizing it cautiously, this may prove to be a disadvantage”* (IDI 17, Female, 23)

#### Individual and social support at the community level

Most students accepted social support from their friends and peers, and a few respondents received social support from their neighbors. The participants felt the social connection that assisted them in reducing loneliness and being alone, notably the respondents with a nuclear family. However, one respondent reported that he was not profoundly connected with any social circle. During the lockdown period, it was his strong family bonds that provided support. But he expressed the importance of social connection outside of family members representing motivations associated with employment:

*“I kept in touch with everyone at my workplace, and my duty seemed to maintain the public relationship. Besides, I enjoyed watching movies and dramas. My social connections were low during the lockdown period. I didn’t talk to anyone except my family members. I didn’t use Facebook and talked less with my friends. Moreover, I experienced some incidents of misunderstanding. However, I tried to increase social connections for job purposes”* (IDI 13, Male, 22)

### Theme 3: Institutional responses

The third theme is institutional responses to the financial and other mental health challenges for students across private and public universities.

#### Financial subsidies and soft loan incentives

Some university authorities provided financial subsidies. Essential financial support to purchase digital technologies such as laptops and smartphones assisted students in public universities in joining online classes during the pandemic. An official of the University Grants Commission indicated that these students received over 5 million USD as soft loans without interest for such purposes (KII 3, UGC official). Additionally, faculty members in public universities sometimes funded selected students from their pockets. This economic assistance worked as a coping for many students during the crisis. However, some students missed these soft loan incentives due to budget limitations.

Institutional support, mainly economic, assisted students in overcoming their hurdles and difficulties during COVID-19. Twenty percent tuition fee waiver, regular scholarships, subsidies on university expenses (inside the campus), and reductions in library fees, student fees, or student activities fees were essential supports provided in private universities. Teachers assisted students in purchasing electronic gadgets, laptops, and smartphones in public universities. Some participants indicated that they received economic support from the university and mental support from teachers during COVID-19. One participant expressed her feelings:

*“We received a specific waiver, we are still receiving it. We did not pay library fees for being absent from offline classes. Our university used to email for counseling, but what’s the point of the email if they pressurize us with work? Our Vice-Chancellor keeps sending us an email to express how we all are going through a tough time, and we love him. His words or those emails are comforting”* (IDI 5, Female, 23)

Unlike private university students, respondents who attended public universities did not receive a tuition fee waiver. However, a few participants stated they received some form of scholarship or financial support from their teachers. Some stated that they received no support from their university. Installment basis support to buy electronic gadgets was available for some students in public universities. Some students were able to get free bus services during EID (an Islamic festival). A public university student addressed his ideas:

*“Students with no smartphone have been asked to apply for financial support. They got USD 80 each on the condition of paying in installments. Semester fees have been waived. Some teachers were very supportive and asked me to contact them personally for any special problem. Moreover, scholarships have been arranged especially for those poor students. Before Eid, 15 to 16 University buses deliver free rides for students to reach their homes”* (IDI 13, Male, 24)

In addressing responses to educational institutions in Bangladesh, an official of the University Grants Commission explained:

*“More than 3 million students and teachers at primary, secondary, and tertiary level received zoom link facilities for online classes across the country. Students in public universities received approximately USD 5000000 as a soft loan with zero interest to buy a laptop or smartphone to attend online classes. Moreover, UGC agreed with five leading mobile companies to provide internet facilities for students from both public and private universities countrywide during the COVID-19 pandemic”* (KII 3, UGC official).

#### Mental health support and counseling

The existing mental health counseling and psychological support system did not function actively in some public and private universities. Some institutions did not have any mental health counseling or psychological support center. A public university participant commented on the support provided by his university:

*“Our University has a Mental Health Counseling Center. Healthcare staff do not provide mental health support with sincerity. A considerable number of students attempted suicides, some of whom committed suicide during this lockdown. Getting an appointment to meet healthcare staff is a great challenge. Sometimes, meeting with the care providers takes a long time than usual that eventually disappoints the university students and officials to utilize mental healthcare support”* (IDI 18, Male, 22)

Another private university participant addressed the referral process for support and the attempts at mental health promotion:

*“Our authority has provided the email address of counselors and psychiatrists. It was confidential. I didn’t go for my mental support. I have congratulated this initiative because it was confidential. Moreover, they also provided links to meditative sound and relaxation, and Zen Music”* (IDI 10, Male, 21)

One university informant indicated the need for improved provision of mental health support within universities, suggesting that universities could use university student psychology students to offer support:

*“We must provide first aid mental health services, including organizing associated referral services. We need to start an integrated hotline service 24/7 so students can access mental health counseling, psychiatric medicine suggestion, and trauma counseling care. We need to allocate a handsome budget for this mental health management”* (KII 2, Psychologist)

The flexibility and motivational support provided by faculty members were beneficial in coping for some students. However, the absence of mental health counseling centers, insufficient care providers, inadequate healthcare services such as limited counseling hours, and the long queue of getting serials to meet healthcare providers at the existing facility center frustrated students during the pandemic. As reported by a KII, *“Mental health support was an auxiliary part before this COVID-19 pandemic. Very few private and public universities tend to provide mental health support. Most universities do not have established mental health counseling centers to offer counseling support. Motivational sessions are insufficient compared to full-time psychological support”* (KII 2, Psychologist). As indicated by psychologist KII, this absence of mental health services is not unique to universities but is a nationwide problem. In Bangladesh, mental health counseling costs more than any other health counseling for the general population. As most universities do not have their own health mental counseling center, students have to seek mental health counseling from outside institutes or service centers which is expensive for them. These difficulties may lead to intensified mental illnesses and suicidal tendencies during COVID-19. The expert opinion demonstrates that *“we must provide first aid service to overcome these mental health challenges. Then, we can refer to the experts according to their problems. It is high time to start an integrated 24 h hotline service to ensure national coverage. Our intern Psychologists and current students from the Department of Psychology can provide mental health services. Public hospitals such as National Mental Health Institution and Dhaka Medical College can contribute and coordinate to support mental health at the national level”* (KII 2, Psychologist).

## Discussion

Our findings of Dhaka, private and public university students identified common mental health challenges that appeared to be affected by academic pressure, sleeping disorders, coupling relationship breaks up, fear of infection, and traumatic experiences during lockdown periods. Impatience and mood swings led students to engage in conflict with their siblings. Social media posts, loss of concentration, financial losses, loss of social connection, cognitive malfunctioning, the health of elderly members, and death of relatives contributed to initiating anxiety, creating stress, and substantially developing depression in students. Financial constraints and learning resource shortages mirrored disproportionately in students. For instance, those students having financial burdens before the pandemic experienced more economic hurdles during the pandemic compared to their fellow cohorts from the affluent family background. Similarly, access to electronic resources such as hardware, software, and the internet, including institutional financial assistance and mental health support reflected unequally in students across public and private universities. Students utilized social support to manage these challenges and adopted multiple personal, family, and community strategies.

Mental health is still not a resourced area of health and well-being in Bangladesh. However, inadequate counseling service hours, long queues of students on the waiting list, online sessional services, absence of university counseling support centers, and insufficient mental health promotion limited healthcare utilization for students. Moreover, referral services to access mental health care utilization appeared as compromising for financial and socio-cultural barriers. Browsing Facebook posts, sharing memes, fear of corona infection, and uncertainty of education life created anxiety in university students aged 19–24. Our findings are consistent with study findings reported from Iran, China, and Bangladesh (6, 10, 35). Financial burdens created excessive pressure for students at the individual and family levels. More specifically, family members losing jobs, curtailed salaries, or closures of businesses at the family level intensified mental stress in students because of their dependence on their family’s income for tuition fees and other educational costs. These results are consistent with findings from France, Canada, and the United Kingdom (7–9). However, COVID-19 associated restrictions such as staying at home, limited outside movements, loss of physical activity, and loss of physical and social connection coupled with burdens of losing family members and relatives challenged the routine life of students. These factors directly affected depression levels in university students in Bangladesh. Similarly, disruptions to physical activity, sleeping disorders, and screen time utilization were largely documented in students compared to the same population just after starting the pandemic (36). The longitudinal data analysis showed a 90 percent increase in depression rates compared to the same population just before the pandemic (36).

Students spent quality time with their family members, and their siblings appeared to fill the friend gap. Besides individual engagement in games and recreational activities, family bonding appeared as a powerful influencer in minimizing stress, anxiety, and depression in students. Our study findings match the research outcomes of university students in Nepal (37). Therefore, the family can be a great supporter and enhance resilience if relationships with the family are already positive and robust. In contrast, if any students experience a toxic family with violence and hardship, their family support may not function as protective of enhancing resilience during the pandemic period. Meanwhile, their pre-existing violent attitudes and adversity may turn out as serious effects to decrease students’ resiliency during the crisis period.

Overall, changes in socio-economic, cultural, and lifestyle factors affected students’ mental health challenges during COVID-19, and these challenges were met by utilizing social support at individual and community levels and institutional support at the university level. Self-sourced support, social support from family members, social connection with close relatives, and institutional financial and mental health support worked as coping strategies to ensure mental health resiliency for students. Given the established strong positive association between social capital and mental well-being (38), our study findings appear similarly consistent with study results in United States, Sweden, China, and Iran during the COVID-19 pandemic (17–21). Students with more social connections and networking showed more resilience to retain their mental health during COVID-19. For instance, social support and social connections act as a mechanism to reduce anxiety, stress, and depression for students. While resilience building is deeply integrated into the social structure, preferably in social connections and embedded in ideological and societal expectations of social groups (25), students with social links experienced more resiliency in terms of mental health and well-being during COVID-19. However, mental health challenge is not still considered an institutionally recognized health problem at the family level in Bangladesh society. Besides financial subsidies and sessional motivational services through social media links, many universities could not establish mental health counseling support centers. A few universities with regular mental health counseling services struggled to cope with the ever-increasing service demand during COVID-19. Compared to economic and mental health support received at the institutional level, self-support, social support at the family level, and social connection with close family members, friends, and relatives at the individual and community level worked more effectively to keep university students psychologically resilient.

Given the underlying study findings, our suggestion testifies that government needs to consider more mental health services such as low-cost or cost-free counseling, traumatic care, and referral healthcare, including organizing mental health promotion campaigns and activities across the country. In this connection, a robust national policy may be designed based on further baseline survey study findings. To avoid self-harm and suicidal attempts cases soon, government health agencies should reconsider identifying and managing acute mental health illnesses of the students and those who suffered from psychiatric disorders before the pandemic. As mental healthcare has not gained formal institutional recognition in family and educational institutions, the system requires a significant change to set up a standardized national mental healthcare support system to promote mental health counseling and care. These initiatives will reduce financial health burdens created by mental health impacts and save many lives. The health ministry may consider an intervention plan to address the needs of students of different backgrounds to intervene in the already-stated services at the country level.

We strongly suggest that universities must figure out the significance of mental well-being for their students. Based on the current study outcomes, universities require organizing more mental health services such as ensuring mental well-being, providing counseling, and suggesting referral services for acute mental illnesses on site. As an educational entity in the country, public and private universities cannot avoid their responsibilities to promote mental well-being and provide mental health services among students. Different socio-cultural groups and university clubs can contribute to raising countrywide awareness about the significance of social support and connections and mental health promotion at the community level. The health ministry can open a mental health helpline 24/7 using a particular app for students. This helpline will help screen initial mental health symptoms and suggest further mental health counseling and care.

## Limitation

Firstly, due to the countrywide COVID-19 restrictions and the nature of the study design, it was difficult to draw a larger sample that significantly limited broader generalization. Secondly, face-to-face interviews were limited in many instances, which made it harder for researchers to draw inferences from participants’ emotional responses and make connections.

## Conclusion

Mental health is still not a resourced area of health and well-being in Bangladesh. Lockdowns, mobility restrictions, financial constraints, academic pressure, coupling relationship breakup, excessive internet dependency, and traumatic experiences unevenly challenged the mental health conditions of students across universities during COVID-19. Multiple support sources at the family and community level, including family bonding and social networking, worked as social support to allow students to cope with anxiety, stress, and depression. Financial subsidies for tuition and development fees, soft loans for purchasing electronic gadgets and mobile data, inadequate mental health promotion, and sessional mental health counseling contributed to minimizing the mental health impacts. Concentration on developing strong social support and improving increased financial subsidies, including learning resources, can be effective. The state should design a rationally justified national intervention plan to engage different healthcare professionals, psychiatrists, intern psychologists, public health experts, sociologists, and university stakeholders. Their consolidated guidelines will assist in establishing mental healthcare support centers at university levels, including a national 24/7 helpline service to avoid immediate and prolonged negative mental health impacts. Future research may focus on concentrating the long term mental health challenges to avoid further serious mental illnesses and suicidal ideation for students across educational institutions.

## Data availability statement

The raw data supporting the conclusions of this article will be made available by the authors, without undue reservation.

## Ethics statement

The studies involving human participants were reviewed and approved by Ethical clearance for the study was issued by the Research Ethics Committee of the East West University Center for Research and Training (EWUCRT) at East West University (Ethical Approval Number: round 13, no 9 (3)/2021). The patients/participants provided their written informed consent to participate in this study. Written informed consent was obtained from the individual(s) for the publication of any potentially identifiable images or data included in this article.

## Author contributions

MB, MT, and SR conceived and designed the study, and supervised the overall project. MB and MT administered the field work and processed data for the analyses. MB, MT, and SA analyzed the data. MB drafted, revised, and edited all the sections of the manuscript. MB and SR critically reviewed the manuscript and added valuable insights. All authors contributed to the article and approved the submitted version.

## Funding

This project has been funded by the East West University Center for Research and Training (EWUCRT), East West University, Dhaka, Bangladesh.

## Conflict of interest

The authors declare that the research was conducted in the absence of any commercial or financial relationships that could be construed as a potential conflict of interest.

## Publisher’s note

All claims expressed in this article are solely those of the authors and do not necessarily represent those of their affiliated organizations, or those of the publisher, the editors and the reviewers. Any product that may be evaluated in this article, or claim that may be made by its manufacturer, is not guaranteed or endorsed by the publisher.
